# Estimation of white matter hyperintensities with synthetic MRI myelin volume fraction in patients with multiple sclerosis and non-multiple-sclerosis white matter hyperintensities: A pilot study among the Indian population

**DOI:** 10.3934/Neuroscience.2023011

**Published:** 2023-05-24

**Authors:** Nisha Syed Nasser, Krish Sharma, Parv Mahendra Mehta, Vidur Mahajan, Harsh Mahajan, Vasantha Kumar Venugopal

**Affiliations:** 1 Mahajan Imaging Private Limited, New Delhi, India; 2 CARPL.AI, New Delhi, India

**Keywords:** synthetic MRI, myelin loss, multiple sclerosis, MAGiC, white matter fraction

## Abstract

**AIM:**

Synthetic MRI (SyMRI) works on the MDME sequence, which acquires the relaxation properties of the brain and helps to measure the accurate tissue properties in 6 minutes. The aim of this study was to evaluate the synthetic MRI (SyMRI)-generated myelin (MyC) to white matter (WM) ratio, the WM fraction (WMF), MyC partial maps performing normative brain volumetry to investigate MyC loss in multiple sclerosis (MS) patients with white-matter hyperintensites (WMHs) and non-MS patients with WMHs in a clinical setting.

**MATERIALS and METHODS:**

Synthetic MRI images were acquired from 15 patients with MS, and from 15 non-MS patients on a 3T MRI scanner (Discovery MR750w; GE Healthcare; Milwaukee, USA) using MAGiC, a customized version of SyntheticMR's SyMRI® IMAGE software marketed by GE Healthcare under a license agreement. Fast multi-delay multi-echo acquisition was performed with a 2D axial pulse sequence with different combinations of echo time (TEs) and saturation delay times. The total image acquisition time was 6 minutes. SyMRI image analysis was done using SyMRI software (SyMRI Version: 11.3.6; Synthetic MR, Linköping, Sweden). SyMRI data were used to generate the MyC partial maps and WMFs to quantify the signal intensities of test group and control group, andcontrol group , and their mean values were recorded. All patients also underwent conventional diffusion-weighted imaging, i.e., T1w and T2w imaging.

**RESULTS:**

The results showed that the WMF was significantly lower in the test group than in the control group (38.8% vs 33.2%, p < 0.001). The Mann-Whitney U nonparametric t-test revealed a significant difference in the mean myelin volume between the test group and the control group (158.66 ± 32.31 vs. 138.29 ± 29.28, p = 0.044). Also, there were no significant differences in the gray matter fraction and intracranial volume between the test group and the control group.

**CONCLUSIONS:**

We observed MyC loss in test group using quantitative SyMRI. Thus, myelin loss in MS patients can be quantitatively evaluated using SyMRI.

## 1. Introduction

Multiple sclerosis (MS) is a chronic immune disease of the brain characterized by formation of lesions in the central nervous system and myelin loss [1]. In MS, there is a loss of oligodendrocytes, resulting in thinning or complete loss of myelin. Over the years, the process of remyelination will take place, and the inability of oligodendrocytes to rebuild the myelin sheath will result in the development of scars like lesions or plaque around the damaged axons [1].

Magnetic resonance imaging (MRI) is the preferred imaging modality for diagnosing MS. Visual assessment of these plaques using contrast-enhanced MRI can be challenging due to subjective sensitivity evaluation in early stages. Although MRI can be used to assess the lesion changes in MS, there is a need to use advanced MRI methods to investigate new MS plaques to allow early diagnosis. Automated assessment of the mean lesion volume, myelin-to-white-matter (WM) ratio and WM fraction (WMF) and comparing them to data obtained from a healthy reference population will provide a reliable and reproducible method that is comparable to the visual assessment [2]. Also, the use of deep learning algorithms to automate the quantification of plaque number, along with the mean lesion volume of myelin loss, can improve the sensitivity and accuracy of MS diagnosis [3].

Synthetic MRI NEURO (SyMRI version: 11.3.6; SyntheticMR, Linköping, Sweden) is a new innovation in the market which has transformed the subjective way of reporting in MRI to quantitative reporting. This software helps to measure the accurate tissue properties of the different tissues in the brain. SyMRI works on the multi-delay multi-echo acquisition (MDME) sequence, which acquires the relaxation properties of the patient in an acquisition time of 6 minutes [4].

The basic concept of the sequence is to work on the relaxation properties of the particular tissues, so it also provides relaxation maps with a color coding scheme, and T1, T2, R1, R2 and PD relaxations maps are provided by the software [5]. These relaxation times can be used for early detection of pathologies, or to see infiltration of any pathology to the adjacent normal tissues. Relaxation maps provided by the software constitute the raw data which is post-processed, and synthetic contrast weighted images are formed. A total of 12 different contrast images are provided, which include the T1w, T2w, FLAIR, proton density weighted (PDw), Diffusion inversion recovery (DIR), Phase-sensitive inversion recovery (PSIR), Short-TI Inversion Recovery (STIR) and other contrast images; with the suppression of WM, gray matter (GM) or fat can be generated in less than 10 seconds [4]. The images are synthetic, so parameters like repetition time (TR), echo time (TE) and inversion time (TI) can also be adjusted as per the requirements.

Brain tissue segmentation i.e., myelin, GM, WM and cerebrospinal fluid (CSF) segmentation, is also provided by the software. The myelin segmentation provided by the software is the first of its kind, and it can help better the visualization of the myelin. Also, automatically quantified reports for the segmentation are provided by the software, which gives information about the accurate volume of particular material in the brain, which includes the intracranial volume (ICV), brain parenchymal volume (BPV), brain parenchymal fraction (BPF) [4].

Quantitative values for each slice are also available in the software; in addition to this, if more information is required on a particular area that can also be acquired by freehand drawing the region of interest (ROI) for that particular region. Another unique feature is the reference curves provided by the software, which helps to compare the particular patient with a normal patient of the same age; it also tells if the patient has normal values or if their values are high or low [4].

The software comes with an easy interface and a lot other features, such as distance measurement, contrast enhancement, freehand ROI, automated ROI and windowing tools [4].

SyMRI Neuro is the only clinical USA Food and Drug Administration (FDA)-approved product available in the market which provides myelin segmentation and myelin estimation, and it easily provides us the myelin details.

This study was planned to be conducted in two phases. In Phase 1, we conducted a pilot study to evaluate the feasibility of SyMRI to detect the myelin loss and WMF loss in MS patients with white-matter hyperintensities (WMH) as compared to the non-MS patients with WMHs. In Phase 2, our aim is to analyze the number of plaques on the conventional MRI and synthetic generated T1w FLAIR images, to compare them with the number of plaques generated by artificial intelligence (AI) outputs and to determine how the mean lesion volume and lesion count can benefit the radiologists in day-to-day clinical practice.

## 2. Materials and methods

### 2.1. Study Population

A retrospective case-control study was conducted in a private diagnostic center to quantify the myelin loss in patients with MS. MRI images were acquired from 15 MS patients with WMHs (mean age = 47.9 years; 7 males, 8 females) (herein referred to as the test group), and from 15 non-MS patients with WMHs (mean age = 43.8 Years; 8 males, 7 females) (herein referred to as the control group). The study was conducted in accordance with the Declaration of Helsinki, 1996. Ethics approval was not required because a waiver was obtained due to the use of retrospective data. Data were collected between March 2021 and March 2022. The patients in this study were scanned for internal research purposes. Written informed consent was obtained from all the patients.

### 2.2. Image Acquisition

All patients underwent MRI scans on a 3T MRI scanner (Discovery MR750w; GE Healthcare; Milwaukee, WI, USA) with a 48-channel head coil. All subjects underwent a fast multi-delay multi-echo acquisition (MDME) sequence called MAGiC, which is a customized version of SyntheticMR's SyMRI® IMAGE software marketed by GE Healthcare under a license agreement. Quantitative SyMRI was performed by applying an MDME sequence using a 2D axial pulse sequence with different combinations of TEs and saturation delay times (TR = 4000 ms, TE1 = 22 ms, TE2 = 89 ms, slice thickness =4 mm, interslice gap = 1 mm, flip angle = 90°, matrix = 320 × 256 and rFOV = 0.8). At our diagnostic center, two TEs and four delay times were used to generate eight real images and eight imaginary ones, which were then used to quantify the longitudinal T1 and transverse T2 relaxation times and the PD. The data acquired at each section were used to produce T1, T2 and PD maps, which were then used to calculate the synthetic magnetic resonance images. The total image acquisition time was 6 minutes. All subjects also underwent conventional diffusion-weighted imaging, T1w FLAIR and T2w imaging. Coronal T2 FLAIR images were obtained using the following parameters: TE = 120.4 ms; TR = 9000 ms; TI = 2248 ms; slice thickness = 5 mm; interslice gap = 5.5 mm.

### 2.3. Data Analysis

White-matter lesions (WMLs) that appear hyperintense/bright on T1w FLAIR images were included in the analysis.

SyMRI image analysis was done using SyMRI post-processing software (SyMRI version: 11.3.6; SyntheticMR, Linköping, Sweden) that automatically and simultaneously generates different synthetic weighted contrasts, i.e., T1w, T2w, PDw, T1w FLAIR, T2 FLAIR, DIR and PSIR images. Also, the SyMRI imaging data were used to automatically segment the total brain volume, including the WM, GM, CSF volume, total ICV and the myelin (MyC) partial maps, along with the R1 and R2 relaxation maps.

The MyC-to-WM ratio was calculated by using SPSS software to quantify the signal intensities of the normal appearing white matter in the test group and control group, and their mean values were recorded. All measurements were performed in a randomized and blinded manner. Also, age-stratified reference curves for the given population were obtained and compared with the normative value, together with data plotting, curve fitting and confidence interval calculation. For each tissue type, data were plotted against the patient age.

Brain tissue measurements were normalized to ICV, yielding the WM fraction (WMF = WM volume/ICV) and GM fraction (GMF = GM volume/ICV).

We hypothesized that there would be myelin loss in the test group, and that this may be revealed with the quantitative synthetic MRI results for myelin quantification. Hence, we used the quantitative synthetic MRI results to investigate the association between myelin partial maps and WM volume.

### 2.4. Statistical Analysis

The data were evaluated for normality by using histograms and a Kolmogorov–Smirnov test; the variables are reported as mean ± SD.

Differences in the means of the WM, GM and CSF volumes, myelin partial fraction map, WM-to-myelin ratio and WMF were assessed using a Mann-Whitney U test.

Statistical analysis was performed using SPSS v22 (IBM Released 2013, Armonk, NY, USA: IBM). The significance level for the tests was set at p < 0.05 (two-sided).

## 3. Results

### 3.1. Demographics of the Participants

Table 1 compares the demographic data of the ages, MyC-to-WM fractions, total myelin volumes and total WM volumes between the test group and control group. The mean age of the test group was 47.93 ± 19.44 years, and that of the control group was 43.8 ± 8.03 years. No significant differences in age between the test and control groups was noted (P = 0.819).

### 3.2. WMF Analysis

Non-parametric independent sample t-tests were used to assess the differences in WMF, WM volume, GMF and ICV between the test group and the control group. The results showed that the WMF was significantly lower in the test group than in the control group (38.8% vs 33.2%, p < 0.001). Interestingly, we noted that the mean WM volume was significantly different between the control and the test group (511.64 ± 91.55 vs 441.6 ± 90.55, p = 0.012). Also, there were no significant differences in GMF and ICV between the test group and the control group (see Table 2).

### 3.3. Myelin Fraction Analysis

Differences in the means of myelin volume fraction and MyC-to-WM fraction were assessed using the Mann-Whitney U nonparametric t-test. We noted a significant difference in the mean myelin volume between the test group and the control group (138.29 ± 29.28 vs.158.66 ± 32.31, p = 0.044).

### 3.4. Grey Matter Volume Fraction Analysis

GMF analysis revealed that there were no significant differences in the mean GM volume fraction (i.e., the GMF) or BPV between the test and the control group.

**Table 1.** Descriptive statistics of the test group and the control group.

**Group**		**N**	**Minimum (mL)**	**Maximum (mL)**	**Mean (mL)**	**Std. Deviation (mL)**
**Control group**	age	15	32.00	63.00	43.8000	8.02852
	WM	15	410.80	721.70	511.6400	91.55785
	GM	15	564.10	789.40	658.6733	65.14459
	CSF	15	77.40	199.40	120.6267	40.80008
	MyC	15	119.50	229.10	158.6600	32.31487
	BPF %	15	87.20	93.40	90.9667	2.12625
	BPV	15	1054.00	1545.00	1192.9333	144.80355
	ICV	15	1149.00	1727.00	1313.4000	177.32609
	WM_MyC	15	3.03	3.69	3.2422	.16493
**Test group**	age	15	17.00	74.00	47.9333	19.44026
	WM	15	319.40	708.50	441.6000	90.55181
	GM	15	542.90	772.00	657.3467	76.49269
	CSF	15	93.30	348.40	203.1067	90.23704
	MyC	15	98.10	217.00	138.2933	29.28072
	BPF %	15	74.90	94.00	84.8600	5.75745
	BPV	15	901.00	1473.00	1124.5333	151.71819
	ICV	15	1006.00	1566.00	1327.5333	170.87082
	WM_MyC	15	2.98	3.47	3.2027	.15909

**Table 2.** Overview of the mean volume fractions of the ICV (GMF, WMF), WM volume (WMV), GM volume (GMV), CSF volume (CSFV) and WM-MyC ratio derived using the Mann-Whitney U test between the test group and the control group.

	**WMV(mL)**	**GMV(mL)**	**CSFV(mL)**	**WM-MyC Ratio**	**WMF (mL)**	**GMF (mL)**	**ICV (mL)**
**Mann-Whitney U**	52.000	112.00	41.00	98.000	32.000	101.000	103.000
**Wilcoxon W**	172.00	232.00	161.00	218.000	152.000	221.000	223.000
**Z**	-2.510	-0.021	-2.966	-0.601	-3.339	-.477	-.394
**Asymp. Sig. (2-tailed)**	0.012*	0.983	0.003*	0.548	.001*	.633	.693
**Exact Sig. [2×(1-tailed Sig.)]**	0.011^b^	1.000^b^	0.002^b^	0.567^b^	.000^b^	.653^b^	.713^b^

*Significant at p < 0.05

**Figure 1.**
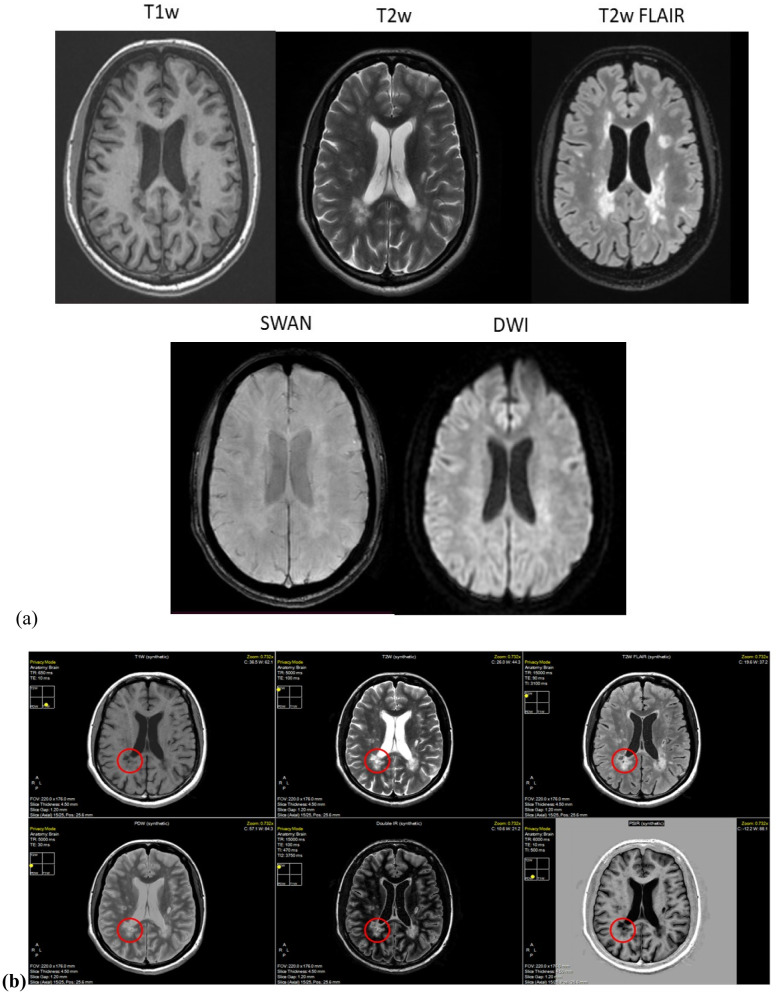
(a) Conventional MRI derived images; (b) Different contrasts generated using the SyMRI software in a 33-year-old female patient with a known case of MS. 69–70 lesions in the supratentorial and seven lesions in the infratentorial regions of the brain were seen on the T2/FLAIR images (min. 3 mm).

**Figure 2.**
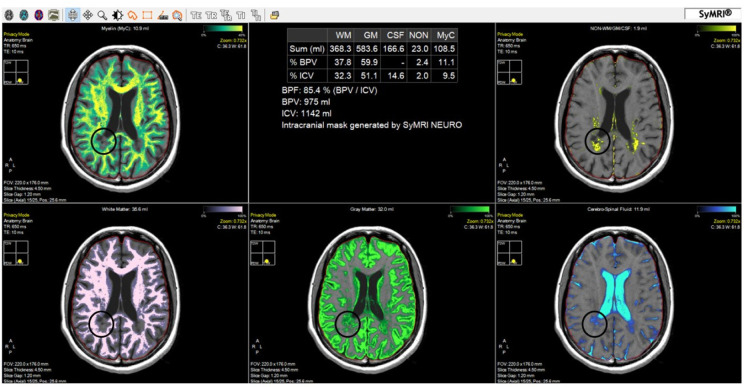
Automatically generated brain segmentation map using the SyMRI software.

## 4. Discussion and conclusion

In this study, we aimed to find the benefit and use of SyMRI software for automatic segmentation of the different types of brain tissue volumes in MS patients. We found that the myelin volume, WM volume and WMF were significantly lower in the test group as compared to the control group. There was no significant difference in the GM volume between both the groups. It is well established in theory that MS primarily affects the WM [1], and we found that to be true in our study as well.

To our knowledge, this is the first study to estimate the WM content in patients with MS by using synthetic MRI in an Indian population. A previous multi-center, multi-reader study has indicated that there is comparable accuracy between synthetic MRI data and conventional MRI on neuroradiology reports [6]. Another study indicated that the contrast ratio is better for synthetic versus conventional DIRs; also, the number of plaques reported for synthetic MRI is larger than that for conventional MRI [3]. Another strength of this study is that we took into account the individual brain sizes in this study by comparing the WMF (with respect to the ICV) and the WM volume. Synthetic MRI has also been found to be faster than conventional MRI, thus saving valuable time for the radiographer and the patient.

However, through this study, we have found that the 6-min MAGiC sequence for automatic segmentation of the brain volume and generation of different contrast images can decrease the workload of the radiologist and the infrastructure requirements for undergoing an MRI scan, as well as increase the efficiency of the system as a whole.

The main limitation of this study is the small sample size. Also, we did not perform any ROI analysis on the scans to look for the exact location of WM loss. Synthetic T2FLAIR is more prone to artifacts than conventional T2FLAIR, which is specifically seen in the posterior limb of the internal capsule [6], or next to the CSF [3]. Few studies have shown that GM might be heavily affected by MS as well [7],[8], but that was not the case with this study.

Although MRI is a sensitive method for detecting the WMLs, we did not assess the correlation between the number of plaques and disease severity.

Though the healthcare industry has been relatively slow in adapting AI into their workflow, deep learning (DL) tools are commercially available for clinical settings. However, adoption of these software packages in everyday clinical practice is limited. For Phase 2 of the study, we are specifically interested in comparing the myelin loss in MS patients to that of non-MS patients, and we would also like to explore the added benefits of using these DL tools for assessment of the quantitative outputs in a clinical setting. These DL tools, along with the SyMRI software, can be useful to quantify the WM, myelin loss and number of plaques in patients with MS. We will also correlate the number of plaques and the amount of WM loss with disease severity or the reproducibility of synthetic MRIs with conventional MRIs.

To conclude, this study shows that SyMRI can be used as an alternative to conventional MRI to generate the different contrasts. Thus, the measurements of WM volume, WMF and myelin volume can be assessed using SyMRI with good reliability and reproducibility.
